# On the Einthoven Triangle: A Critical Analysis of the Single Rotating Dipole Hypothesis

**DOI:** 10.3390/s18072353

**Published:** 2018-07-20

**Authors:** Gaetano D. Gargiulo, Paolo Bifulco, Mario Cesarelli, Alistair L. McEwan, Hossein Moeinzadeh, Aiden O’Loughlin, Ibrahim M. Shugman, Jonathan C. Tapson, Aravinda Thiagalingam

**Affiliations:** 1 The MARCS Institute, Western Sydney University, Milperra, NSW 2214, Australia; h.moeinzadeh@westernsydney.edu.au (H.M.); j.tapson@westernsydney.edu.au (J.C.T); 2 Department of Electrical Engineering and Information Technology (DIETI), “Federico II” The University of Naples, 80100 Naples, Italy; pabifulc@unina.it (P.B.); cesarell@unina.it (M.C.); 3 School of Electrical and Information Engineering, University of Sydney, Sydney, NSW 2006, Australia; alistair.mcewan@sydney.edu.au; 4 School of Medicine, Western Sydney University, Campbelltown, NSW 2650, Australia; aiden.oloughlin@gmail.com; 5 Cardiology Department, Campbelltown Hospital, Campbelltown, NSW 2650, Australia; shugmano@hotmail.com; 6 School of Medicine, University of Sydney, Sydney, NSW 2006, Australia; aravinda.thiagalingam@sydney.edu.au

**Keywords:** basic cardiology science, ECG, Wilson Central Terminal, Einthoven triangle

## Abstract

Since its inception, electrocardiography has been based on the simplifying hypothesis that cardinal limb leads form an equilateral triangle of which, at the center/centroid, the electrical equivalent of the cardiac activity rotates during the cardiac cycle. Therefore, it is thought that the three limbs (right arm, left arm, and left leg) which enclose the heart into a circuit, where each branch directly implies current circulation through the heart, can be averaged together to form a stationary reference (central terminal) for precordials/chest-leads. Our hypothesis is that cardinal limbs do not form a triangle for the majority of the duration of the cardiac cycle. As a corollary, the central point may not lie in the plane identified by the limb leads. Using a simple and efficient algorithm, we demonstrate that the portion of the cardiac cycle where the three limb leads form a triangle is, on average less, than 50%.

## 1. Introduction

Modern electrocardiography (ECG) is still based upon the simplified assumption that the electrical activity of the heart can be reduced to that of a single electrical dipole rotating around a fixed point in the chest, the projection of which constitutes the so-called 12-lead ECG on the direction identified by pairs of electrodes [1]. This assumption was formulated in the 1930s by F.N. Wilson, who introduced the so-called precordials, or chest leads. In modern electrocardiography, precordials are voltage measurements. Having said that, at the time, precordials were measured as the current circulating into a circuit composed of one out of six pre-defined positions on the chest, and an estimation of the fixed point around which the equivalent heart dipole is supposed to rotate during the cardiac cycle [1]. Wilson himself defined this reference point as the average of the Einthoven limb electrodes: Left Arm (LA), Right Arm (RA), and Left Leg (LL), whereas the average is simply operated by connecting the three electrodes to a single common point via three identical, “high value” resistors [1].

As one may note, the Right Leg (RL) is not included in the original electrical activity of the heart model [1,2,3]. This is because the original instrument used to demonstrate the limbs’ ECG by Einthoven was an extremely sensitive galvanometer (Ampere-meter) [1,4]. Therefore, also the limbs’ ECG at the time was, by definition, a measure of the tiny current impressed from the heart to the limbs. Because there is no direct current pathway between the RL and LL that includes the heart, the circulating current is measured only between the arms, and between each arm and the LL.

**Figure 1:**
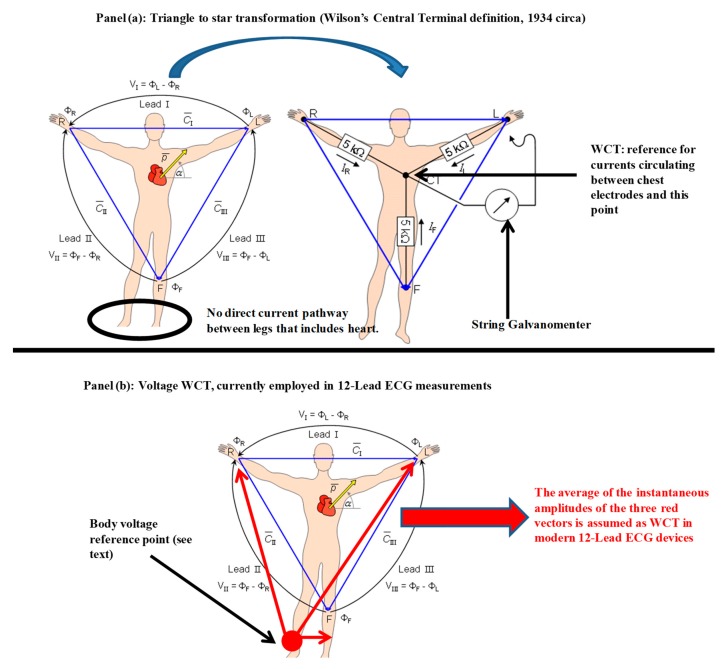
Traditional VS modern definition of WCT: Panel (**a**): Einthoven’s triangle (**left**) and Wilson’s transformation (**right**); Panel (**b**): modern voltage WCT.

In summary, it is possible to conclude that ECG recordings were originally a measure of the net current impressed by the electrical activity of the heart circulating into an external circuit closed by the measurement instrument. With this assumption, it was natural for Wilson to complete the transformation from the triangle (Einthoven’s triangle) to the equivalent star circuit when he faced the problem of finding a reference for his chest leads (see Figure 1 panel (a)). In theory, if each of the Einthoven leads measures the net current impressed by the heart between two limbs, averaging all the electrodes together should give the best approximation of the point of origin: the neutral point of the cardiac electrical activity. In honor of his measurements and experiments, this reference terminal was named after him (Wilson’s central terminal, or simply WCT), and the term ‘unipolar’ was introduced for precordial leads.

In this framework, with this study we will demonstrate the following research hypothesis:

Voltage measurement of limb leads, although measured in a closed circuit, does not form a triangle for the majority of the duration of a cardiac cycle; hence, a centre and centroid that constitute the WCT cannot be identified, as per Wilson’s hypothesis.

We prove this research hypothesis by analysing a total of 599 12-lead ECG recordings that include 549 recordings taken from the PTB database (freely available of Physionet [5]), and 50 recording taken the pilot study for our ECG device which is capable of recording the components of the WCT [2,6]. Using these recordings, we first assess where the limb leads form a triangle, and calculate the inner angles. Assessment of WCT amplitude is performed only for the 50 recordings where this is available.

## 2. Materials and Methods

For this paper, we employed a total of 599 ECG recordings. The first 549 were taken from the online Physionet databank [5] PTB database [7], while the remaining 50 were selected from one of our recent studies [2]. All subjects volunteered for our study and gave written consent (this study was approved on 23 September 2015 by the National Human Ethics Committee of Liverpool Hospital with the protocol number HREC/15/LPOOL/302).

This method section is divided into three parts. In the first part, we introduce the theoretical background and principles of physiological measurements of Voltage/Current for electrocardiography that form the context of our research hypothesis; in the second, we delve into the details of the equilateral triangle hypothesis which is at the base of the standard ECG theory (this is used to demonstrate the study hypothesis); in the third and the final parts of this method section we give a summary of the hardware used for the 50 recordings where WCT components are available.

During the work for this paper, we produced a very large amount of processed data and figures. As we aim to make our research entirely reproducible, the necessary processing scripts are included, together with the results of the processing for each single dataset we employed as additional material. The new data that we recorded during our clinical evaluation [2] are also available for download in Matlab format upon request.

### 2.1. Methodology Background: Principles of Voltage/Current Measurement in Electrocardiography

Before delving into the full explanation of our method, we must mention that Wilson’s hypothesis and the related triangle to star transformation has been challenged at both the theoretical [8,9,10,11,12,13] and practical level [2,3,6,10,11,13,14,15,16,17,18,19,20,21] several times in the past 80 years, with several researchers confirming that, contrary to what Wilson’s principal postulates [1,22,23], the amplitude of the WCT can be comparable to (or even larger than) the limb leads, and can exhibit variability during the cardiac cycle similar to any other ECG signal [2,8,10,16,24,25,26,27,28]. Still, as already mentioned, one may note that the original instrument that both Einthoven and Wilson used to formalize the triangular ECG model and the WCT was measuring the current impressed by the heart to the body surface, while modern electrocardiography uses voltage amplifiers [28]. Therefore, it is possible to evince that, contrary to the original definition of WCT, in modern ECG devices the WCT used is the instantaneous average of the voltages of the three limbs (RA, LA, and LL) relative to a reference voltage (see Figure 1 panel (b)) [3,17].

Moreover, once again we must stress the consequences of exchanging voltage measurements for current ones, as this difference is crucial for our method. Although it is clear that there is a well-known relationship of cause and effect between the voltage potential measured between two points of a circuit and the current circulating in the circuit itself, one must not forget that voltages are relative measurements performed by differential reading with respect to a reference point assumed to be neutral (or steady). Even the so-called “true differential” electronic amplifiers require a reference point to operate correctly [29,30,31]. In biomedical applications, particularly ECG, this reference is created virtually by the internal circuitry (i.e., voltage supply bootstrap [32]), and one may have the impression that the amplifier works off the two points of connection in a true differential manner, i.e., this is only an impression, as the virtual reference voltage “moves” within the power supply rails according to the estimated common mode. This solution is often employed in single-lead ECG designs. In multiple-lead designs, this reference point is a true reference point on the body, and usually this additional connection is used also to re-inject a part of the measured signal inside the body to increase the signal-to-noise ratio at certain frequencies (i.e., power line frequencies). In both cases, the selected reference point on the human body is placed at the RL, and the circuitry designed to re-inject the signal inside the body is known as the Right Leg Driver (RLD), or Driven Right Leg (DRL) [32,33,34,35]; for this reason, in Figure 1, panel (b), the reference point for voltage measurements is noted on the RL [3,17].

**Figure 2:**
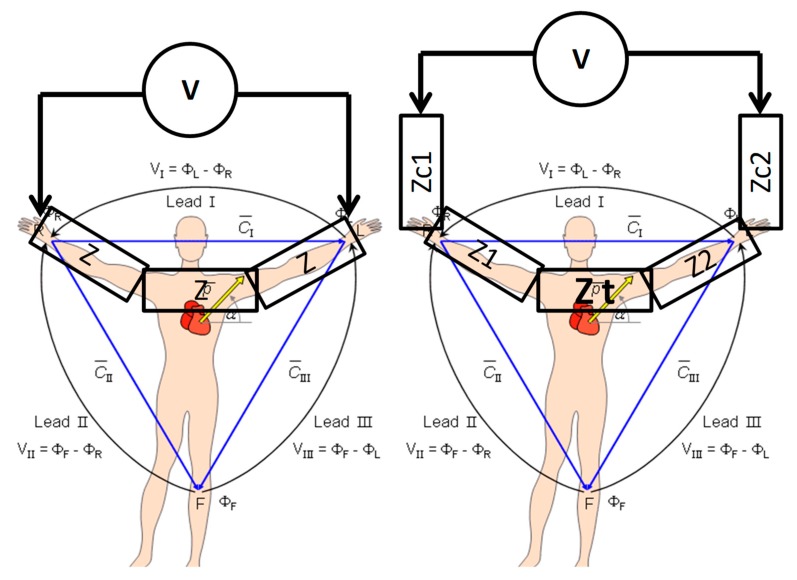
Direct comparison of idealized (**left**) measurement of lead I as voltage VS real measurement (**right**) that takes into account contact impedances (Zc1 and Zc2), different limbs impedance (Z1 and Z2) and the variable torso impedance (Zt) which changes rhythmically following the respiration.

Due to the natural link between voltage and current, and to the impractical use of sensitive current-measuring devices, cardiologists became used to referring to all measurements pertaining to ECGs in mV, neglecting the fact that measuring voltages rather than current implies having to deal with the different impedances of body sections. Taking Ohm’s law into account, which states that the voltage is the product of current and resistance (Voltage = Resistance × Current), each lead can be interpreted as the voltage drop across a composed resistance (impedance, as a matter of fact) due to the net current impressed by the heart to the points of measurement. As an example, lead I (see Figure 2) can be interpreted as the voltage drop across the sum of the contact impedance at both electrodes, the impedance of each arm, and the variable impedance of the chest (due to respiration) across the shoulder.

Of course, evoking the simplifying hypothesis that the body is a homogeneous volume conductor, and hence, limb electrodes are all placed at an equal distance on a homogeneous conductor (constant resistance), with no (or negligible) contact impedance aside from a simple constant proportion between the values of current and voltages, these are perfectly interchangeable. However, in real life recordings, this is not always the case, and often, impedance/contact impedance imbalance between ECG electrodes is not verified, or is fixed. Impedances which are not purely resistive or homogeneous can alter the phase relationship between voltage and current, adding a delay variable with frequency; this may affect the limb leads which are measured across different portions of the torso that change in shape with respiration and body posture. Altered phase relationships may also affect raw limbs’ potential to form the WCT. Because RA, LA, and LL potentials are first measured as voltage potential difference between the reference point (RL) and the other limbs’ electrodes, different body impedances and contact impedances may impose different delays upon the signals, thus affecting the final WCT shape in an unpredictable manner [3,17].

### 2.2. Study Hypothesis: On the Equilateral Triangle Hypothesis

If the three cardinal leads are indeed on a plane and form a triangle (even if not equilateral), in order to obtain a WCT which is at least at the centroid of the triangle (center coincide with centroid only in equilateral triangles), the three cardinal limbs need to satisfy the basic condition of a triangle. This is because, under the original Einthoven/Wilson assumption, the equivalent electrical activity of the heart is entirely projected into the geometrical plane identified by the limbs [1,4,36]. In other words, the ECG values need to satisfy the so-called triangular inequality which states that the sum of any two of the three lengths that are candidates for the triangle must be greater than the third. If this is satisfied, it is then possible to calculate the inner angles.

In order to prove this hypothesis with a computationally efficient method, we assumed that at every point of the cardiac cycle, the three cardinal leads form a triangle, and we calculated the area using Heron’s formula:

(1) $$Area=\sqrt{p\left(p-a\right)\left(p-b\right)\left(p-c\right)}$$

where *a*, *b* and *c* are the three measurements of the sides and *p* indicates half of the perimeter:

(2) $$p=\frac{a+b+c}{2}$$

Obviously, if *a*, *b*, and *c* do not form a triangle, the area calculated using (1) is either null or a complex number. We proceeded to calculate the inner angles only for those terns of points constituting a triangle or, in other words, where (1) gives a positive result. To our calculation script, we passed the cardinal leads following their cardinal order (I, II and III), and received the calculated angles with the following order: ‘opposed to lead I’, ‘opposed to lead II’, and ‘opposed to lead III’.

Because the ECG has some brief iso-potential segments (i.e., following the p-wave), we expected some percentage of points within each cardiac cycle where (1) is not satisfied (i.e., the same iso-potential value for all three leads), as well as some iso-potential segments between beats. While there is nothing that we can do to remove the iso-potential segment between ECG characteristic waveforms, we can remove the one between beats, limiting the calculation to one single cardiac cycle. According to the existing literature, flat iso-potential segments between characteristic waveforms can account for up to 40% of the length of a single beat [1]. Therefore, it would be normal to find (1) satisfied only in roughly 60% of the points for each cardiac cycle. Namely, we expect (1) to have a solution only during the QRS, p-wave, and T-wave. Furthermore, because noise, particularly baseline drift/wandering, could alter the ECG, we removed such noise using a zero phase lag non-causal filter with a cut-off frequency at 0.6 Hz. To minimize the effect of artifacts and power-line noise, the signals are also filtered using an array of zero phase lag non-causal notch filters at power-line and harmonics frequencies, together with a low pass filter at 149 Hz. For each of the 599 recordings, a single beat was selected manually from the cleaned signal. We attempted to select a beat with the following characteristics:

High signal-to-noise ratio: no muscles/movements artifacts
Avoid pacemaker where possible
Avoid multiple p-waves in case of atrial flutter
Avoid ectopic where possible.

Once the selected beat is isolated, Equation (1) is applied to each tern of points corresponding to each of the signal samples. The percentage of points where (1) is satisfied, together with the length of each beat, is stored in a separate variable, and the inner angles are calculated only where (1) is satisfied (non-null real value).

### 2.3. A Brief Summary of the Hardware Used

**Figure 3:**
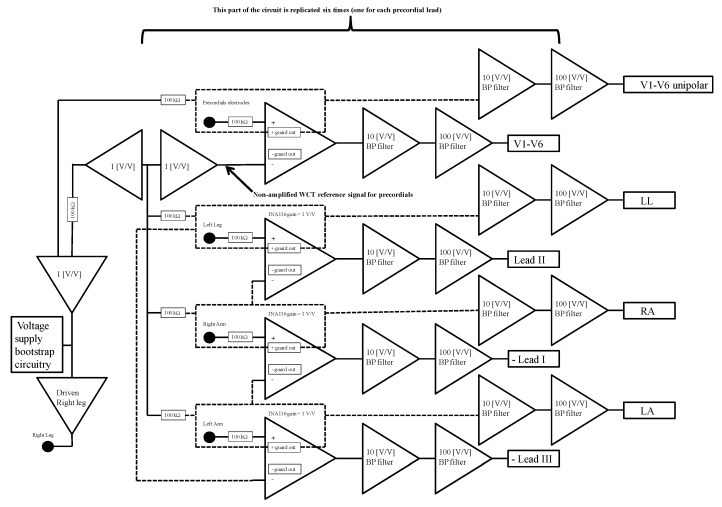
Block diagram of the employed hardware (adapted from [2]).

Full details of the hardware we used to record the data can be found in our previous publications. In particular, the general description of the true unipolar recording channel can be found in [2]. We want to highlight that our hardware can also record true unipolar precordials (see Figure 3). In other words, similar to what we have done to record the WCT components, we record and store the voltage potential of each of the precordial electrodes as directly referred to the RL. This allows us to calculate new precordials, i.e., to subtract in the post-process a reference potential different from the WCT. Coherence between the true unipolar leads and 12-lead ECGs is ensured by the extremely high correlation between the recorded 12-lead signal and the calculated 12-lead signal. In other words, using lead *I* as an example, we ensured that:

$$Correlation\left(\left[RA-LA\right],I_{12lead}\right)>98\%$$

As shown in the block diagram of Figure 3, each exploring electrode is connected to the non-inverting input of one instrumentation amplifier. For our design we selected the INA116 by Texas Instruments. This peculiar amplifier includes a special buffer for each input (see the dashed rectangles around each electrode connection in Figure 3) that allows direct measurement of the electrode potential, signal duplication [2], as well as input shielding [2,30].

The signal supplication feature is directly employed for the limb electrodes to create direct limb leads. For instance, as it is possibly to infer from Figure 3, once duplicated by the buffer inside the INA11,6 the Left-Leg electrode signal is connected to the last instrumentation amplifier on the diagram to create Lead III. In this way, due to our design choice of connecting electrodes only to non-inverting inputs, some leads (i.e., Limb III) output negative values. The negative leads are multiplied by −1 by the recording software [2]. The complete circuit uses a voltage supply bootstrap and a Driven Right-Leg circuitry (calibrated to contain leakage current below 100 µA) to minimize noise pick-up and increase the signal to noise ratio [2,4,32].

## 3. Results

**Figure 4:**
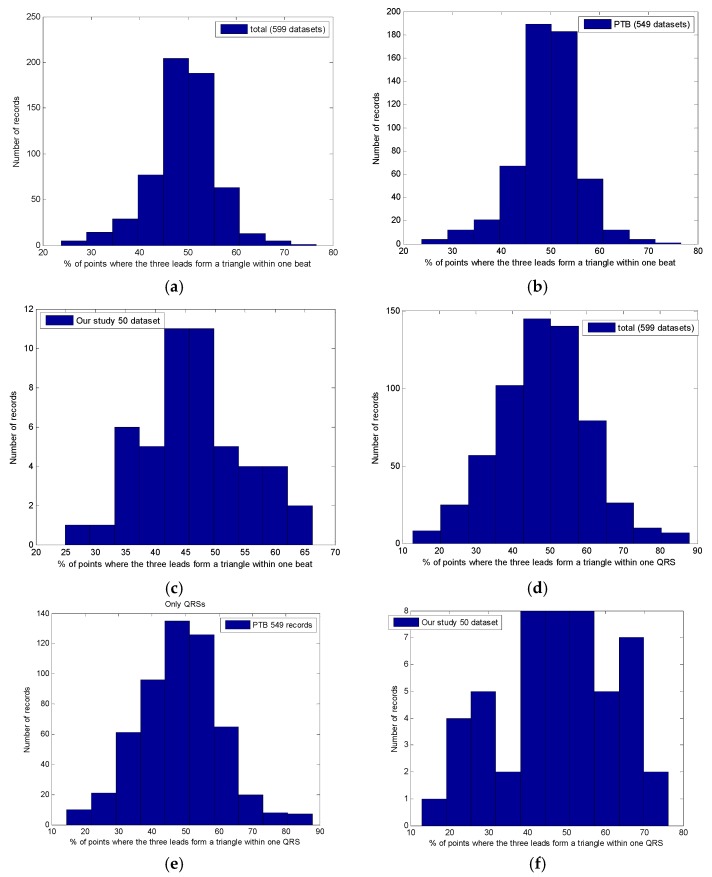
Percentage of points where Equation (1) is satisfied within one beat (panels a to c) and one QRS (panels d to e): (**a**) total, mean 49%; (**b**) PTB database, mean 49%; (**c**) our study only, mean 46%; (**d**) total, mean 48%; (**e**) PTB database, mean 48%; (**f**) our study only, mean 47%.

As expected, Equation (1), when applied to a single beat for all 599 datasets, produced correct results only for a fraction of the points. In Figure 4, we present the detailed histograms of the distribution of the percentage of points within each beat that satisfy (1). For this measurement we accepted (considered satisfied) any small positive result obtained by application of (1); in other words, we compared directly with zero. In Figure 4a, we depict the general histogram for all 599 datasets; histograms for the PTB dataset alone, and for fifty recordings constituting our Dataset 2, are shown in Figure 4b,c respectively. The averages where (1) is satisfied are 49% (STD = 12) for the total, 49% (STD = 11) for the PTB database and 46% (STD = 15) for Dataset 2 (values rounded to the nearest integer, interpolations with normal distribution have been implemented using the statistical fitting tool included with Matlab^®^). As can be inferred from the histograms, for the majority of the datasets, the percentage of points where (1) is satisfied is below 50%, which is below what should be expected (~60%) from the theory.

For this reason, we restricted our investigation to the QRS only, where there are no iso-electric segments. From the application of Equation (1) to the QRS only (see Figure 4 panels d to e), we found that, similar to what we observed for the full beat, Equation (1) is satisfied on average for only 48% of the points composing the QRSs with even larger standard deviations. In detail, the averages where (1) is satisfied are 48% (STD = 22) for the total, 48% (STD = 21) for the PTB database, and 47% (STD = 26) for our study.

**Figure 5:**
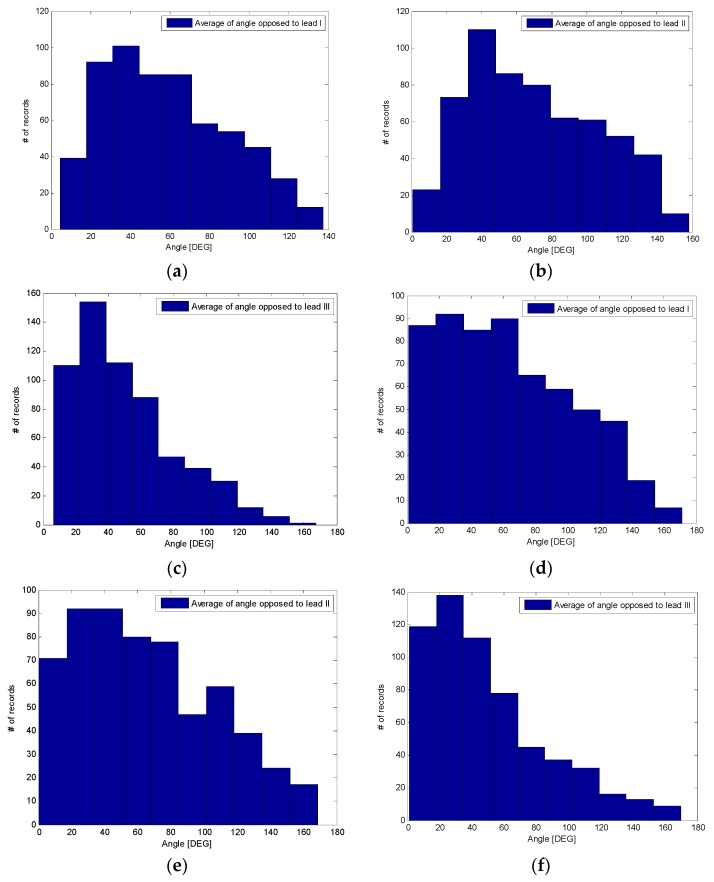
Angle opposed to cardinal leads where Equation (1) is satisfied within one beat (panels a to c) and within one QRS (panels d to f): (**a**) opposed to lead I, mean 59 deg; (**b**) opposed to lead II, mean 70 deg; (**c**) opposed to lead III, 51 deg; (**d**) opposed to lead I, mean 64 deg; (**e**) opposed to lead II, mean 66 deg; (**f**) opposed to lead III, mean 50 deg.

When we looked at the inner angles, although their values (in average) are quite close to the theoretical value of 60 deg, particularly for the QRS (see Table 1), the standard deviations are very high. Full histograms of the inner angles for the full beat, and one single QRS, are depicted in Figure 5; as it is possible to infer from the histograms, the distributions of the bins are very wide, with many bins with similar height.

**Figure 6:**
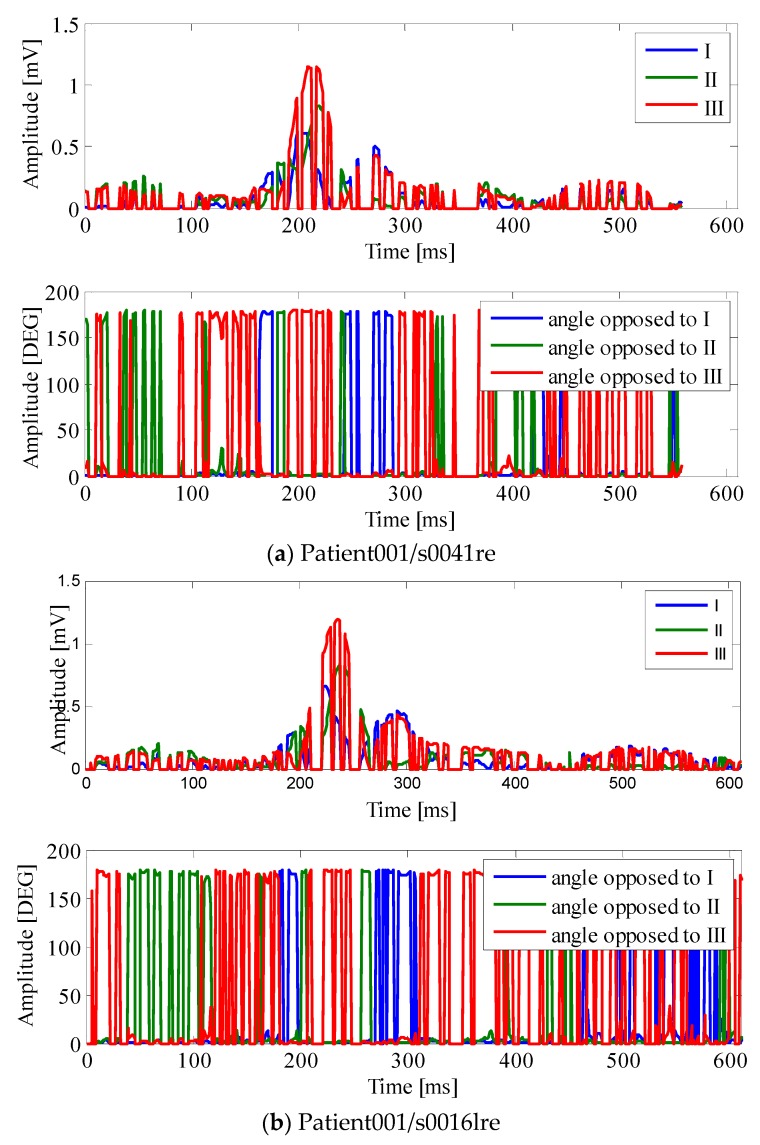
Patient001 recordings on different days. In each panel top: ABS of leads I, II, and III only plotted at the point where Equation (1) gives a non-null real result; Bottom: inner triangle angles plotted only where Equation (1) gives a non-null real result (see text).

An example of how the points where Equation (1) is satisfied distribute in one single cardiac cycle is given in Figure 6. In Figure 6, the points within the cardiac cycle where the absolute value of the three leads do not form a triangle have been “zeroed”; as it is possible to observe from the figure, these sections also affect the QRS region. These seem to be due to a specificity of the patient, and do not vary much in different recordings of the same patient; please compare Figure 6, panel b with the panel belonging to the same PTB database patient recorded on different days. One might also observe that the inner angles of the triangle vary a lot during the cardiac cycle and the QRS (please compare bottom traces of the Figure 6 panels). This suggests that the triangle, when closed, changes shape during the cardiac cycle, and in some cases, it can become quite “open”, with one of the angles exceeding 100 deg. Once again, as it is possible to see from Table 2, there is a lot of variability around the mean for the inner angles.

The amplitude of WCT relative to lead II has been measured for this study too. On average, the WCT amplitude is 78% of lead II, with a standard deviation of 42%. These results are discussed further in the following section.

## 4. Discussion

The small percentage of points where Heron’s formula is satisfied (Equation (1)) suggests that the voltage potential of the CT can actually exceed the cardinal leads in the module, as this point may be located external to the triangle, and may even be outside the geometrical plane identified by the limb electrodes. This may explain why we found peak-to-peak amplitudes for the WCT larger than those reported in the literature in our previous study [2,6]. For this work, in light of our findings, we re-evaluated the peak-to-peak amplitude of the WCT (we also added several patients to our database). However, this time, we measured the amplitude of the WCT at its largest feature and in one beat only, i.e., the one used for the triangle evaluation. The full summary of our measurements is reported in Table 2, and as can be inferred from the table, for a few patients the WCT amplitude is almost double that of lead II.

**Figure 7:**
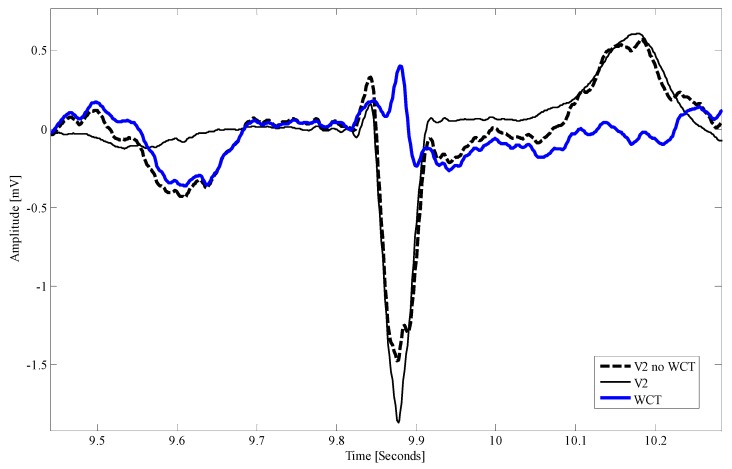
Direct comparison among lead V2 (12-lead ECG), V2 recorded without WCT (bold dashed trace) and WCT (blue bold trace).

Since the introduction of the 12-lead ECG into medical practice, practitioners have learned how to interpret the signals and make diagnoses based on the results of WCT. For this reason, it is extremely difficult to formulate study hypotheses that, while backed up by a suitable statistical body of evidence, can unveil the extent of the implications that the use of a signal this large (and laying on a different geometrical plane) as a reference for precordials has on the practice. However, as it is possible to observe in Figure 7, the WCT influences the precordials. For instance, in this case, while the QRS complex results are slightly larger in the lead V2 (see peak to peak amplitude of dashed bold black trace versus thin black trace) when WCT is subtracted from the v2 electrode, one can easily see that the P-wave is greatly affected (almost erased from the lead V2). Suppression of waveforms, as well as waveform alteration, can have severe implications for the diagnosis of cardiac diseases. For instance, as it is possible to observe from Figure 7, the ST segment is also affected by the WCT, which superimpose a ST depression to V2. ST segment alterations are normally linked to cardiac ischemia or other myocardium illnesses [36]. For this reason, we are still collecting data with our device in a hospital setting, in order to carefully assess the relationship between WCT and cardiac diseases.

## 5. Conclusions

We presented a critical study of the triangular model of the ECG. Although we now have to carefully assess the clinical relevance of our findings, we particularly need to build a solid database of recordings where diagnoses are evaluated by comparing standard 12-lead ECG precordials and precordials obtained using the new personalized CT. We conclude that the WCT introduced for current recordings, due to its large amplitude (sometimes larger than cardinal leads), should not be used as a reference for precordials. Furthermore, our study assessment, based upon a simple method to evaluate when three lengths form a triangle, shows that voltage measurements of cardinal leads fail to form a triangle for the largest part of the duration of a single cardiac cycle, even when calculation is restricted to the QRS complex only, suggesting that the so called ‘Central Terminal’ may actually be outside the geometrical plane identified by the cardinal leads.

## Figures and Tables

**Table 1:** Summary of inner angles.

Angle	Opposed to Lead I [deg]		Opposed to Lead II [deg]		Opposed to Lead III [deg]	
	Mean	STD	Mean	STD	Mean	STD
**One beat**	59	31	70	36	51	30
**One QRS**	64	40	66	41	50	37

**Table 2:** Summary of WCT and cardinal leads measurements (see text).

Patient Record Number	Lead II Amplitude [mV]	Lead I Amplitude as % of Lead II	Lead III Amplitude as % of Lead II	WCT Amplitude as % of Lead II	Age [years]	Gender
1	1.02	118	152	117	70	F
2	0.48	333	283	95	53	M
3	1.55	87	82	77	80	M
4	0.52	240	285	174	85	M
5	1.19	126	43	45	69	F
6	0.83	105	113	52	78	M
7	0.46	199	235	120	73	M
8	0.94	160	121	57	55	F
9	0.20	202	176	114	52	F
10	0.88	158	170	60	70	F
11	1.12	57	115	82	79	M
12	0.58	217	125	56	51	F
13	0.30	123	88	45	52	M
14	1.28	71	46	59	88	F
15	0.77	176	100	30	68	F
16	0.45	222	153	101	49	M
17	0.78	180	122	60	67	M
18	0.70	246	170	206	60	F
19	0.99	105	21	24	54	F
20	0.86	104	32	29	58	M
21	1.13	78	134	63	56	M
22	0.42	136	111	53	70	M
23	1.35	107	78	42	55	F
24	1.18	134	97	38	76	F
25	0.65	81	46	37	74	F
26	0.60	331	385	151	53	M
27	0.87	126	67	26	48	M
28	0.86	177	168	74	57	M
29	0.67	300	267	88	44	M
30	1.81	41	90	56	59	M
31	0.62	144	67	21	84	F
32	0.92	76	133	70	70	M
33	0.66	287	256	79	58	M
34	0.92	141	168	91	51	M
35	0.55	98	193	110	66	F
36	0.41	108	127	117	96	M
37	0.57	170	108	82	53	M
38	0.55	130	159	80	59	M
39	1.06	55	56	50	61	M
40	0.38	136	62	64	68	F
41	0.70	193	106	58	52	F
42	0.92	115	88	49	61	M
43	0.30	115	126	144	70	M
44	1.29	65	55	52	53	F
45	0.32	194	226	173	69	M
46	0.68	299	221	73	47	M
47	0.92	62	150	107	82	F
48	0.61	107	66	33	71	F
49	1.60	24	106	60	73	M
50	0.67	137	120	147	77	M
**Means**	0.80	148	133	78	64.48	
**STDs**	0.36	75	75	42	12.19	
**M = 60%**						
